# A Novel ALDH1A1 Inhibitor Targets Cells with Stem Cell Characteristics in Ovarian Cancer

**DOI:** 10.3390/cancers11040502

**Published:** 2019-04-08

**Authors:** Nkechiyere G. Nwani, Salvatore Condello, Yinu Wang, Wendy M. Swetzig, Emma Barber, Thomas Hurley, Daniela Matei

**Affiliations:** 1 Department of Obstetrics and Gynecology, Northwestern University, Chicago, IL 60611, USA; Nnwani@northwestern.edu (N.G.N.); yinu.wang@northwestern.edu (Y.W.); wendy.swetzig@northwestern.edu (W.M.S.); emma.barber@northwestern.edu (E.B.); 2 Department of Obstetrics and Gynecology, Indiana University, Indianapolis, IN 46202, USA; salvcond@iu.edu; 3 Department of Biochemistry and molecular Biology, Indiana University, Indianapolis, IN 46202, USA; thurley@iupui.edu; 4 Melvin and Bren Simon Cancer Center, Indianapolis, IN 46202, USA; 5 Robert H Lurie Comprehensive Cancer Center, Chicago, IL 60611, USA; 6 Jesse Brown VA Medical Center, Chicago, IL 60612, USA

**Keywords:** ovarian cancer, ALDH1A1, CM37, cancer stem cells

## Abstract

A small of population of slow cycling and chemo-resistant cells referred to as cancer stem cells (CSC) have been implicated in cancer recurrence. There is emerging interest in developing targeted therapeutics to eradicate CSCs. Aldehyde-dehydrogenase (ALDH) activity was shown to be a functional marker of CSCs in ovarian cancer (OC). ALDH activity is increased in cells grown as spheres versus monolayer cultures under differentiating conditions and in OC cells after treatment with platinum. Here, we describe the activity of CM37, a newly identified small molecule with inhibitory activity against ALDH1A1, in OC models enriched in CSCs. Treatment with CM37 reduced OC cells’ proliferation as spheroids under low attachment growth conditions and the expression of stemness-associated markers (*OCT4* and *SOX2*) in ALDH+ cells fluorescence-activated cell sorting (FACS)-sorted from cell lines and malignant OC ascites. Likewise, siRNA-mediated *ALDH1A1* knockdown reduced OC cells’ proliferation as spheres, expression of stemness markers, and delayed tumor initiation capacity in vivo. Treatment with CM37 promoted DNA damage in OC cells, as evidenced by induction of γH2AX. This corresponded to increased expression of genes involved in DNA damage response, such as *NEIL3*, as measured in ALDH+ cells treated with CM37 or in cells where *ALDH1A1* was knocked down. By inhibiting ALDH1A1, CM37 augmented intracellular ROS accumulation, which in turn led to increased DNA damage and reduced OC cell viability. Cumulatively, our findings demonstrate that a novel ALDH1A1 small molecule inhibitor is active in OC models enriched in CSCs. Further optimization of this new class of small molecules could provide a novel strategy for targeting treatment-resistant OC.

## 1. Introduction

Ovarian cancer (OC) has the highest fatality rate of any gynecologic cancer [1]. Although OC is considered a chemo-responsive tumor with very high initial response rates to standard platinum-based therapy [2], most women eventually develop recurrence, which rapidly evolves into resistant disease and is incurable [2]. One of the critical factors contributing to relapse is persistence of quiescent cancer cells that are not eliminated by chemotherapy. It has been speculated that these residual cells are cancer stem cells (CSCs), which can subsequently differentiate and generate recurrent tumors. CSCs have been isolated from OC cell lines, malignant ascites, and primary tumors [3,4,5]. CSCs are characterized by the expression of specific cell surface markers and the ability to self-renew, differentiate, and generate tumors when injected in small numbers into NOD/SCID mice. In culture, CSCs grow as spheres, are able to differentiate into cell subtypes with different phenotypes, and have been implicated in tumor heterogeneity, tumor dormancy, and resistance to chemotherapy and radiation [6]. Several surface markers have been proposed for ovarian CSC identification, including CD44+/CD117+, CD44+/MyoD, CD133+, CD133/ALDH+, or ALDH+. CD44/CD117+ cells are resistant to chemotherapy, generate tumors in immunodeficient mice and display activation of recognized stem cell pathways (e.g., *nanog*, *nestin*, *notch*) [5]. CD44+/MyoD+ cells generate tumors, grow as spheroids and are highly resistant to chemotherapy [7]. CD133+ cells are highly resistant to platinum [8], while ALDH+ cells are tumorigenic in vivo, express stem cell restricted transcription factors, form spheres in non-adherent cultures, and are chemoresistant [4,9,10]. Double positive ALDH+/CD133+ cells are highly tumorigenic and chemo-resistant and represent a rare cell subpopulation within tumors [4]. Here, we focused on the properties of ALDH+ cells and new modalities to target them.

The highly conserved aldehyde dehydrogenase (ALDH) family includes 19 enzymes involved in the metabolism of reactive aldehydes [11]. Through their detoxification functions, ALDHs exert cytoprotective roles in various tissues [12]. In addition, the enzymes catalyze retinol oxidation to retinal, a limiting step during the synthesis of retinoic acid, which activates an important cellular differentiation pathway. Recent reports have linked ALDHs, and particularly ALDH1A1, to stem cells, both in normal tissues, such as the hematopoietic milieu [13], as well as in malignancy [14]. It remains unknown whether the enzyme is only a marker for stem cells or whether it is functionally implicated in maintaining their characteristics. While several other markers have been proposed over the years for identifying ovarian CSCs [5], ALDH1A1 activity detectable through the Aldefluor assay has been validated by several groups, and appears to be a robust phenotype [4,10,15]. The percentage of ALDH+ cells in high grade serous ovarian cancer (HGSOC) cell lines varies between 0.2% to 10%, and ALDH expression is higher in mucinous and endometrioid cancers compared to serous carcinomas [16]. Furthermore, ALDH positivity in OC has been correlated with worse patient survival [16,17]. An enrichment in ALDH+ cells was observed after treatment with platinum both in cells lines and xenografts [18,19], supporting the hypothesis that post-therapy tumors are enriched in cells with progenitor characteristics.

In recent years, emerging efforts have focused on developing new therapies targeting CSCs [20]. Various strategies have been pursued, including agents which block pathways selectively activated in stem cells [21] or which target specific markers expressed on the surface of CSCs. As high ALDH activity appears to be a hallmark of ovarian CSCs [22], development of inhibitors for this family of enzymes is garnering interest [23,24]. We have previously reported that CM37, a small molecule inhibitor for ALDH1A1 discovered from a high-throughput screen of a diverse chemical library, was a potent inhibitor of OC cell proliferation as spheres but had modest effects on differentiated cells [25]. Here, we describe in further detail the effects of this selective inhibitor in ovarian cancer models and the significance of the ALDH1A1 enzyme to the ovarian cancer stemness phenotype.

## 2. Results

### 2.1. CM37 Reduces ALDH Activity and Cell Proliferation in OC Cells

**Figure 1:**
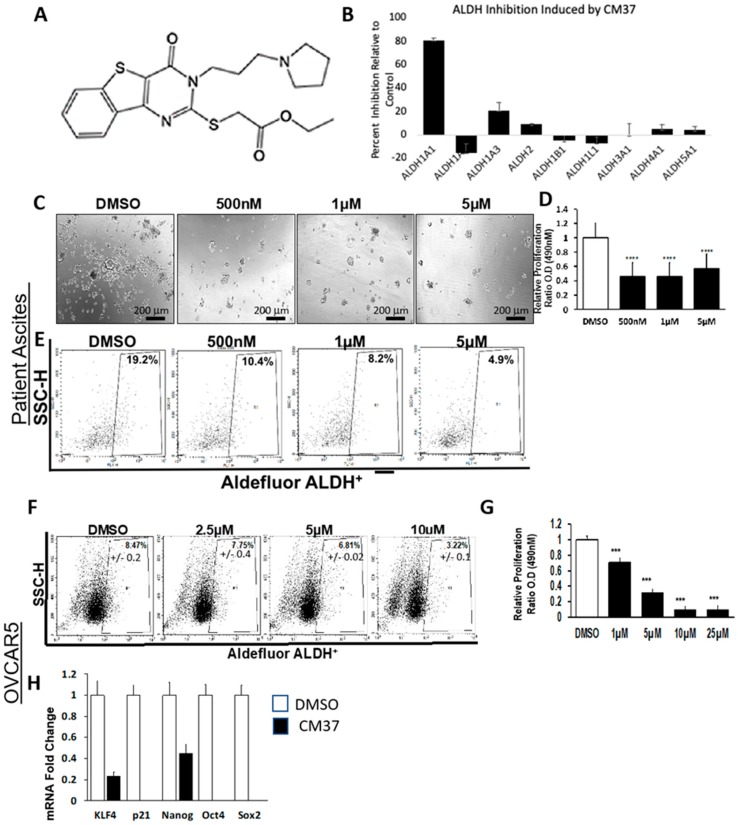
Effects of CM37 on ovarian cancer (OC) sphere formation and stemness markers. (**A**) The chemical structure of CM37; (**B**) percent inhibition of aldehyde-dehydrogenase (ALDH) enzymatic activity by 20 µM CM37 measured in vitro for the different orthologues; (**C**) spheres derived from primary OC cells isolated from ascites fluid and treated with control or increasing doses of CM37 were photographed with an inverted microscope at 100× magnification. (**D**) Numbers of live cells growing as spheres were assessed by CCK-8 colorimetric assay in patient-derived OC cells. (**E**) Percentage of ALDH+ cells in untreated/or CM37-treated (500 nM–5 µM) patient-derived OC cells. (**F**) Percentage of ALDH+ cells in untreated/or CM37-treated (2.5–10 µM) OVCAR5 cells. (**G**) OVCAR5 cells were plated under low attachment conditions for six days; numbers of live cells were assessed by using the CCK8 colorimetric assay. (**H**) Relative expression of stem cell markers *KLF4*, *Nanog*, *Oct4*, *Sox2* as measured by qRT-PCR in ALDH+ FACS-sorted OVCAR5 cells treated with CM37 (1 µM) for 24 h. Bars represent averages of triplicate measurements; **** corresponds to *p* < 0.0001; *** corresponds to *p* < 0.001.

CM37 is a small molecule with a molecular weight of 431.6 Daltons, good predicted drug properties, and no structural similarity to any other aldehyde dehydrogenase inhibitors [26]. The structure of CM37 is shown in Figure 1A. CM37 is a selective and competitive inhibitor of ALDH1A1 with a K_i_ of 300 nM [25]. The drug has minimal inhibitory effects for ALDH1A2, ALDH1A3, ALDH2, or ALDH3A1 at concentrations up to 20 µM [25] (Figure 1B). To investigate its effects on spheroid formation and proliferation, we measured cell viability in FACS-sorted ALDH+ patient-derived OC cells treated with increasing doses of CM37 or control (Figure 1C). A colorimetric CCK8 assay revealed that CM37 significantly reduced the number of live cells in spheroids at all doses (*p* < 0.0001, Figure 1D). To measure its inhibitory activity for ALDH, flow cytometry analyzed Aldefluor enzymatic activity in CM37-treated malignant ascites-derived cells. While 19.2% of vehicle-treated cells displayed high ALDH activity, CM37-treated primary OC cells displayed reduced percentages of ALDH+ cells: 7.6%, 10.4%, 8.2%, and 4.9% after treatment with 100 nM, 500 nM, 1 μM, and 5 μM CM37, respectively (Figure 1E). These results were recapitulated in the HGSOC cell line, OVCAR5. While 8.4% of DMSO-treated OVCAR5 cells exhibited high ALDH activity, a dose-dependent decrease in the ALDH+ population was observed after treatment with CM37 (Figure 1F). A colorimetric CCK8 assay demonstrated that cell proliferation as spheres was significantly blocked by the ALDH inhibitor; starting at the concentration of 1 µM (*p* < 0.001, Figure 1G). Furthermore, the expression of markers associated with stem cell phenotype was tested in ALDH+ OVCAR5 cells treated with 1 µM CM37 for 24 h. CM37 treatment caused a 5- (*p* = 0.002) and 2-fold (*p* = 0.03) decrease in *KLF4* and *NANOG mRNA* expression levels, respectively, while *CDKN1A*/*p21*, *OCT4*, and *SOX2 mRNA* levels were undetectable in CM37-treated cells compared to control treated cells (Figure 1H).

**Figure 2:**
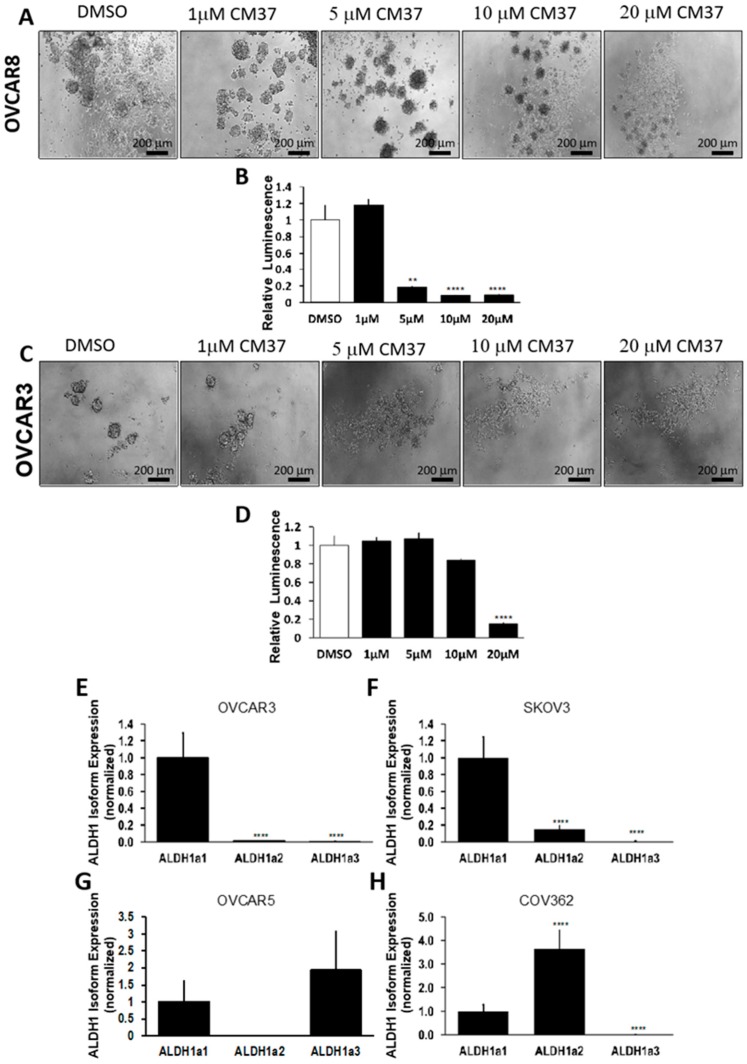
Effects of CM37 on OC sphere formation: CM37 disrupts ALDH1A1-mediated sphere formation and growth under low attachment conditions. (**A**,**B**) OVCAR8 cells were treated with DMSO or 1–20 µM CM37 for six days, and numbers of live cells were assessed by quantifying ATP production via Cell-Titer Glo assay. Spheres were photographed with an inverted microscope at 100× magnification. (**C**,**D**) OVCAR3 cells were treated with control or 1–20 µM CM37 for six days, and numbers of live cells were assessed by quantifying ATP production by using the Cell-Titer Glo assay. Spheres were photographed with an inverted microscope at 100× magnification. (**E**–**H**) Relative expression of ALDH1A isoforms in OVCAR3, SKOV3, OVCAR5, and COV362 cells grown as spheres as measured by qRT-PCR. Bars represent averages of triplicate measurements; ** corresponds to *p* < 0.01; **** corresponds to *p* < 0.0001.

The effects of CM37 on OC cell proliferation cultured as spheres were confirmed in other representative HGSOC cell lines, such as OVCAR8 and OVCAR3. At concentrations ranging from 5 to 20 µM, CM37 significantly blocked sphere formation and ATP production measuring live cells in spheroids derived from OVCAR8 cells (*p* < 0.001; Figure 2A,B). While sphere disruption induced by CM37 was observed by phase contrast microscopy in OVCAR3 cells at concentrations ≥5 µM (Figure 2C), ATP production measuring live cells was decreased only at 20 µM concentration of CM37 (*p* < 0.0001; Figure 2D).

Given the observed differences in sensitivity to CM37 between the tested OC cell lines and the known selectivity of CM37 to ALDH1A1, which is hypothesized to play a key role defining ovarian cancer stemness, we measured the relative abundance of ALDH1 isoforms in the main cell lines utilized in this study. We observed that ALDH1A1 was the predominantly expressed isoform in OVCAR3 and SKOV3 cells (Figure 2E,F), ALDH1A1 and ALDH1A3 were abundantly expressed in OVCAR5, and ALDH1A2 was predominant in COV362 cells (Figure 2G,H).

### 2.2. Characterization of ALDH1A1-Depleted Ovarian Cancer Cells

**Figure 3:**
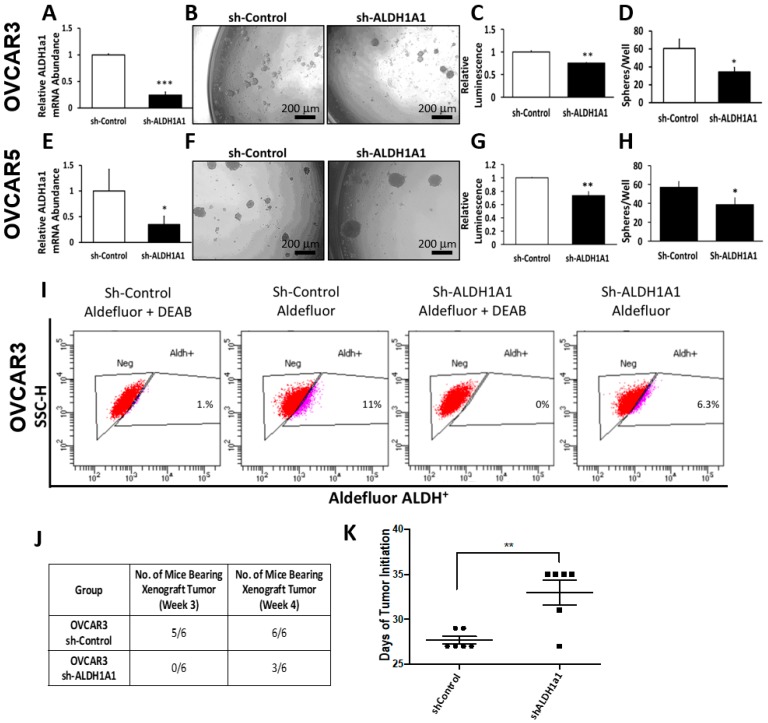
Effects of ALDH1A1 knock down on stemness phenotype. (**A**) OVCAR3 cells were transfected with nontargeting shRNA (Sh-Control) or shRNA targeting *ALDH1A1* (sh-ALDH1A1), and *ALDH1A1* knockdown was verified by qRT-PCR. (**B**,**D**) Sphere formation in OVCAR3 cells stably transfected with sh-Control and sh-ALDH1A1 and plated under low attachment conditions for six days. Spheres were photographed (100× magnification, **B**); numbers of live cells were assessed by using the Cell-Titer Glo assay (**C**), and the numbers of sphere per well were counted (**D**). (**E**) OVCAR5 cells were transfected with sh-Control or sh-ALDH1A1 and assessed for *ALDH1A1* knockdown by qRT-PCR analysis. (**F**–**H**) OVCAR5 cells were plated under low attachment conditions for six days; spheres were photographed (100× magnification, **F**); numbers of live cells were assessed by using the Cell-Titer Glo assay (**G**), and the numbers of sphere per well were counted (**H**). Bars represent averages of triplicate measurements; *** corresponds to *p* < 0.001; ** corresponds to *p* < 0.01; * corresponds to *p* < 0.05. (**I**) Percentage of ALDH+ cells in sh-Control or sh-ALDH1A1 transfected OVCAR3 cells. (**J**) OVCAR3 sh-Control and sh-ALDH1A1 were subcutaneously injected into the flanks of nude mice and tumor initiation was assessed; data captures the total number of tumors detectable at week #3 and at week #4. (**K**) Time to tumor initiation measured in days after subcutaneous injection of sh-Control and sh-ALDH1A1 transfected cells in the flanks of nude mice (*n* = 6 per group).

To determine the functional role of ALDH1A1 in the maintenance of stem cell-like characteristics, we knocked-down *ALDH1A1* in OC cell lines. Lentiviral-mediated *ALDH1A1* depletion resulted in a 4.5-fold reduction in *ALDH1A1 mRNA* expression levels in OVCAR3 cells (Figure 3A). *ALDH1A1* down-regulation reduced the number of spheres and attenuated ATP production corresponding to numbers of live cells in OVCAR3 spheroids (Figure 3B–D). Likewise, targeted depletion of ALDH1A1 in OVCAR5 cells resulted in a 2.5-fold decrease in *mRNA* expression levels (Figure 3E), which led to reduced numbers of spheres and ATP production corresponding to numbers of cells proliferating in spheres (Figure 3F–H).

Flow cytometry assessed ALDH activity in OVCAR3 cells stably transduced with sh-control and sh-ALDH1A1; 11% of sh-control cells exhibited high ALDH activity while 6.3% of sh-ALDH1A1 cells exhibited high ALDH activity, consistent with a reduction in enzyme levels, and perhaps in stemness characteristics (Figure 3I). Of note, ALDH1A1 depletion in OVCAR3 cells was associated with 2.3-fold increase in *ALDH1A3 mRNA* levels (Supplementary Figure S1A), while OVCAR5 sh-ALDH1A1 cells showed a 2.6- and 1.8-fold increase in *ALDH1A3* and *ALDH3*, respectively (Supplementary Figure S1B). These data suggest that significant compensation may exist between ALDH isoforms to maintain the necessary pool of cellular aldehyde dehydrogenase activity.

To further evaluate the significance of ALDH1A1 to cancer stemness, we sought to assess the effects of ALDH1A1 knock down to the tumor initiation capacity (TIC) of OC cells. For this, 20,000 OVCAR3 sh-control or sh-ALDH1A1 cancer cells were injected in the flanks of immune compromised female nude mice. At three weeks, five of the six mice injected with sh-control cells developed detectable tumors (e.g., visible tumors >2 mm in greatest dimension), while none of the mice injected with sh-ALDH1A1 developed tumors. At four weeks, all of the mice injected with sh-control cells had detectable tumors, however only 50% of mice injected with sh-ALDH1A1 OVCAR3 cells developed tumors >2 mm (Figure 3J). The median time to tumor initiation defined as the time between tumor cell inoculation and formation of detectable tumors was 27 days for sh-control cells and 33 days for sh-ALDH1A1 OVCAR3 cells (*p* = 0.003; Figure 3K), suggesting that this isoform plays a functional role in OC cell tumorigenicity. Although tumor initiation from sh-ALDH1A1 transfected cells was delayed compared to control cells, there was no significant difference in tumor size at the final endpoint.

**Figure 4:**
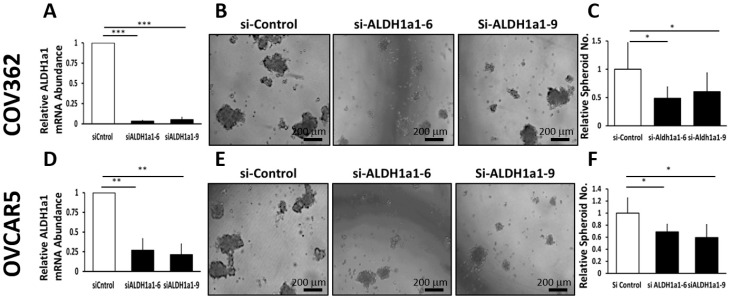
Effects of ALDH1A1 depletion on sphere formation. (**A**) COV362 cells were transfected with 50 nM scrambled siRNA (si-Control) or siRNA targeting *ALDH1A1* (si-ALDH1A1 sequences #6 and #9); *ALDH1A1* knockdown was assessed by qRT-PCR. (**B**) COV362 were plated under low attachment conditions for six days; spheres were photographed under 100× magnification. (**C**) The numbers of spheres per well were counted (five fields per well); graph depicts fold-change. (**D**) OVCAR5 cells were transfected with 50 nM si-Control or si-ALDH1A1 siRNA; cells were assessed for ALDH1A1 knockdown by qRT-PCR analysis. (**E**) OVCAR5 cells were plated under low attachment conditions for six days; spheres were photographed under 100× magnification. (**F**) The number of spheres per well were counted (five fields per well); graph depicts fold-change between cells transfected with control and ALDH-targeting siRNA. Bars represent averages of triplicate measurements; *** corresponds to *p* < 0.001; ** corresponds to *p* < 0.01; * corresponds to *p* < 0.05.

The effects of ALDH1A1 downregulation were further studied in other OC cells transiently transfected with *ALDH1A1*-targeting siRNAs. Transient transfection of siRNAs sequences targeting ALDH1A1 mediated effective down regulation of the enzyme in COV362 and OVCAR 5 cells (Figure 4A,D). *ALDH1A1* knockdown significantly reduced spheroid formation under low attachment conditions in COV362 (Figure 4B,C) and in OVCAR5 cells (Figure 4D–F), as measured by microscopy and sphere counting, supporting the significance of this enzyme to spheroid proliferation.

### 2.3. CM37 Causes Increased DNA Damage in OC Cells

**Figure 5:**
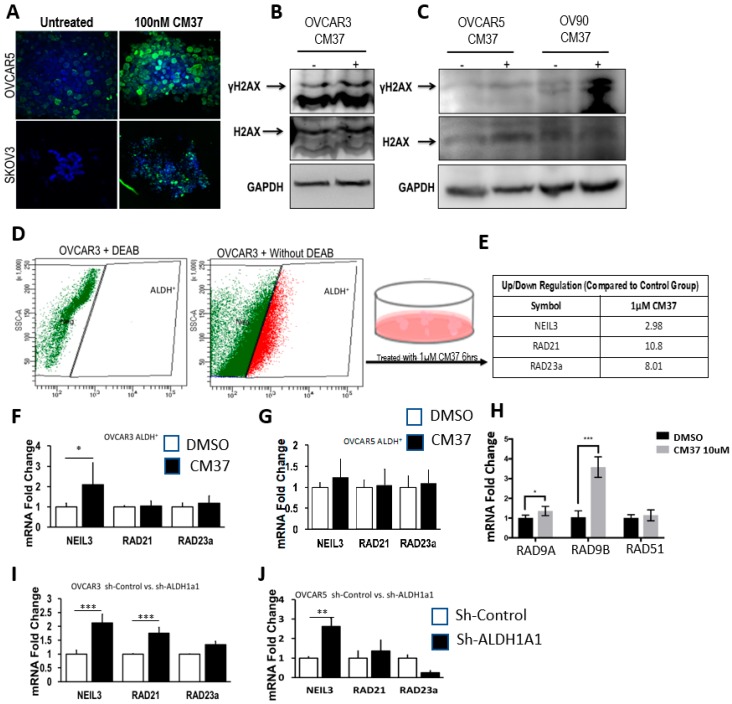
CM37-induced DNA damage response in ovarian cancer cell lines. (**A**) Immunofluorescent staining illustrates increased γH2AX abundance in OVCAR5 and SKOV3 cells cultured as spheres and treated with 100 nM CM37 for 72 h; 60× magnification. (**B**,**C**) Western blot demonstrating increased γH2AX protein levels in OC cell lines cultured as spheres and treated with CM37 for 45 min. (**D**,**E**) OVCAR3 ALDH+ cells were treated with 1 µM CM37 for 6 h prior to measurement of genes implicated in DNA damage response. (**F**,**G**) qRT-PCR measured expression of *NEIL3*, *RAD21*, *RAD23 mRNA* expression levels in ALDH+ OVCAR3 and OVCAR5 cells treated with 1 µM CM37. (**H**) qRT-PCR measured expression of *RAD9A*, *RAD9B*, *RAD51 mRNA* expression levels in OVCAR5 cells treated with 10 µM CM37. (**I**,**J**) QRT-PCR measured *mRNA* expression levels of *NEIL3*, *RAD21*, and *RAD23* in OVCAR3 and OVCAR5 cells stably transduced with sh-Control or sh-ALDH1A1 lentiviral particles. Bars represent averages of triplicate measurements; *** corresponds to *p* < 0.001; ** corresponds to *p* < 0.01; * corresponds to *p* < 0.05.

Several members of the aldehyde dehydrogenase family have been associated with cellular processes that minimize the impact of reactive oxygen species (ROS), which in turn are involved in oxidative stress-induced DNA damage. To elucidate the potential mechanism by which ALDH1A1 inhibition blocks cancer cell proliferation as spheres, we assessed whether CM37 induced DNA damage. CM37-treated OVCAR5 and SKOV3 exhibited increased DNA damage, as evidenced by robust induction of γH2AX, measured through immunofluorescence (IF) staining (Figure 5A). Consistent with these data, western blot analysis confirmed CM37-mediated induction of γH2AX in OC cells. The inhibitor caused robust γH2AX induction within 45 minutes in OV90, OVCAR5, and OVCAR3 cells grown as spheres (Figure 5B,C). To further analyze DNA damage response induced by this inhibitor, ALDH+ FACS-sorted OVCAR3 cells were treated with 1 μM CM37 for 6 h, and a DNA damage-focused RT^2^ PCR array was used to measure *mRNA* transcript levels of a panel of known genes related to DNA damage response and repair (Figure 5D). Among the genes included on this array, CM37 induced a 2.98-, 10.8-, and 8.01-fold increase in the *mRNA* transcripts of *NEIL3*, *RAD3*, and *RAD21A*, respectively (Figure 5E). QRT-PCR analysis of ALDH+ FACS-sorted OVCAR3 cells treated with 1 μM CM37 confirmed a 2-fold (*p* = 0.02) increase in *NEIL3 mRNA* levels, although *RAD21* and *RAD23A mRNA* levels were not significantly changed (Figure 5F). However, no significant change in *NEIL3*, *RAD23*, or *RAD21A mRNA* expression levels was observed in ALDH+ FACS-sorted OVCAR5 cells treated with 1 μM CM37 (Figure 5G). Other DNA damage response genes (*RAD9A* and *RAD9B*, but not *RAD51*) were upregulated in response to treatment with CM37 in OVCAR5 cells grown as spheroids (Figure 5H). We then sought to determine whether *ALDH1A1* knockdown in OVCAR3 and OVCAR5 cells recapitulated the effects of CM37 on *NEIL3, RAD21*, and *RAD23A mRNA* expression levels. ALDH1A1 depletion in OVCAR3 cells resulted in a 2.65-fold (*p* < 0.0001) and 1.95-fold (*p* < 0.0001) induction in the expression of *NEIL3* and *RAD21*, however *RAD23A mRNA* expression levels were not changed. Conversely, *ALDH1A1* depletion in OVCAR5 cells resulted in a 2.6-fold (*p* = 0.003) upregulation in *NEIL3 mRNA* levels, but *RAD21* and *RAD23A mRNA* levels were not significantly changed (Figure 5I,J). Collectively, these data suggest that the novel ALDH1A1 inhibitor CM37 and *ALDH1A1* knockdown induced modest to moderate DNA damage response in OC spheroids.

### 2.4. CM37 Induces ROS in Ovarian Cancer Cell Lines

**Figure 6:**
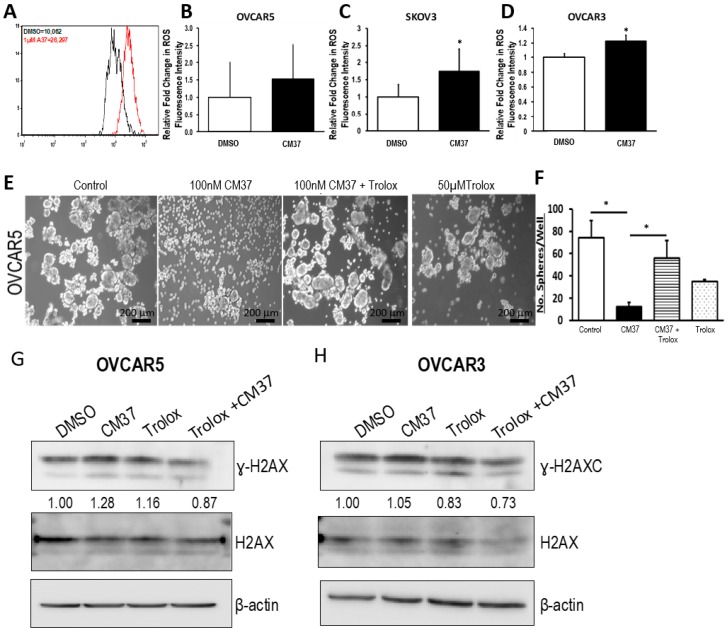
CM37 increases reactive oxygen species (ROS) levels in OC cells. (**A**) Representative flow cytometry for total intracellular ROS levels in OVCAR5 cells treated with DMSO or 1 µM CM37. (**B**–**D**) Fold change in ROS levels measured by flow cytometry in SKOV3, OVCAR3, and OVCAR5 treated with DSMO or 1 µM CM37 for 1 h. (**E**,**F**) Sphere formation in OVCAR5 cancer cells treated with 100 nM CM37 +/− 50 µM trolox for three days. Spheres were photographed under an inverted microscope (**E**) and counted (five fields per well; (**F**). Bars represent averages of triplicate measurements; * corresponds to *p* < 0.05. (**G**,**H**) Western blot measures γH2AX, H2AX, and β-actin protein levels in OVCAR5 and OVCAR3 cells cultured as spheres for six days and treated with 5 µM CM37 for 45 min, after 1 h or not of pre-treatment with Trolox (20 µM).

Knowing that ALDH participates in the regulation of the intracellular redox, and considering that reactive oxygen species (ROS) are inducers of DNA damage, we evaluated the effects of CM37 on intracellular ROS levels in OC cell lines. OC cell suspensions were treated with 1 µM CM37 or DSMO for 60 minutes before measurement of intracellular ROS through flow cytometry. CM37 treatment resulted in elevated ROS production in OVCAR5 cancer cells, although the increase did not reach statistical significance (*p* = 0.06) (Figure 6A,B). However, treatment with 1 µM CM37 resulted into a significant 1.2-fold (*p* = 0.005), and 1.74-fold (*p* = 0.02) ROS elevation in OVCAR3 and SKOV3 cell suspensions, respectively (Figure 6C,D). These data led us to hypothesize that by blocking ALDH1A1, CM37 treatment increases intracellular ROS levels, which in turn cause DNA damage, hindering cell survival and proliferation. To evaluate this possibility, we evaluated spheroid formation in OVCAR5 cells treated with CM37 in the presence or absence of Trolox (6-hydroxy-2,5,7,8-tetramethylchroman-2-carboxylic acid), a ROS scavenger. Spheroid formation was significantly reduced in OVCAR5 cells treated with 100 nM CM37 (*p* = 0.017) and this effect was reversed in cells pre-treated with 50 µM trolox (*p* = 0.05) (Figure 6E,F). Furthermore, the mild induction of γH2AX caused by treatment with CM37 and measured by western blotting in OVCAR5 and OVCAR3 cells was attenuated by pre-treatment of the cells with 20 µM Trolox for 1 h (Figure 6G,H). Collectively, these data support the concept that CM37 is involved in the regulation of intracellular oxidative stress.

## 3. Discussion

Our data provide proof of principle support for the concept that ALDH inhibitors block stemness and cell proliferation in OC models, and back further development of such small molecules for targeting and eradicating CSCs. We show that CM37, a selective and potent ALDH1A1 inhibitor [25], effectively inhibits OC cell proliferation as spheres, induces accumulation of intracellular ROS, and inflicts DNA damage. These effects were consistent between cell lines and primary cancer cells sorted from malignant ascites. Similar effects were observed following knockdown of the *ALDH1A1* isoform, supporting its significance to the maintenance of stemness characteristics. However, functional redundancy among isoforms in this family and the distinct patterns of expression of isoforms in various models or cell lines may limit the activity of highly selective inhibitors, suggesting that drugs with a broader spectrum of inhibitory activity might induce more potent anti-CSCs results. Our findings have several implications.

Aldefluor activity has been increasingly recognized as a marker of cells with progenitor/stemness characteristics in OC [22], but also in other cancer models [13,24]. There are 19 genes encoding various aldehyde dehydrogenases with similar functions and tissue specific patterns of expression. The main catalytic activity of ALDHs is to oxidize aldehydes generated from cellular metabolism through an NAPD(P)-dependent reaction. However, ALDHs participate in other cellular processes, including the regulation of retinoid signaling by conversion of retinal into retinoic acid. This role places a spotlight on ALDH as a key regulatory node in the process of cellular differentiation. Indeed, ALDH inhibition by DEAB was shown to delay cytokine-induced differentiation of hematopoietic progenitor cells through direct inhibition of retinoic signaling in these progenitors [27].

ALDH1A enzymes are expressed abundantly in the liver, pancreas, intestine, and hypophysis and are present variably in cancer, particularly in colon, liver, pancreatic, and some ovarian and breast tumors [17]. Among the various isoforms, ALDH1A1, 1A2, and 1A3 have been shown to be expressed and active in cancer [28] and have been implicated in the development of chemo-resistance [17,25], a key characteristic of CSCs. Evaluation of specific isoforms in cancer tissue is somewhat limited by the specificity of available antibodies. Additionally, ALDH expression is considered to be restricted to progenitor cells, a rare cell population within tumors, further limiting the ability to distinguish between isoforms. The available literature supports that ALDH1A3, ALDH3A2, and ALDH7A1 isozymes are expressed more abundantly in ovarian tumors, particularly in mucinous and endometrioid type tumors [16]. Our current results support the significance of ALHDH1A1 isoform to proliferation of OC cells as spheres and maintenance of a CSC phenotype, but also show that other ALDH1A isoforms are expressed in OC cells and could compensate for selective inhibition or knock down of ALDH1A1.

ALDH1 activity is increased in OC cells growing as spheroids compared to monolayers [18,25] and in OC cells and tumors that survived exposure to platinum [18,19]. Recent data from our group and others demonstrate that platinum activates cancer-associated fibroblasts in ovarian xenografts, thereby stimulating IL6 secretion, which in turn, transcriptionally upregulates ALDH activity [19]. These observations suggest that a potential application of ALDH inhibitors could occur after completion of standard platinum-based therapy, with the goal of eradicating residual, chemotherapy-resistant, or tolerant cells, which are enriched for ALDH activity and responsible for giving rise to recurrent, recalcitrant tumors. Other strategies which could indirectly block ALDH1A1 include agents targeting transcriptional regulatory mechanisms, such as bromodomain and extra terminal (BET) inhibitors [29], DNA hypomethylating agents [18], and STAT inhibitors [19] which have been shown to eliminate ALDH+ cells residual after platinum treatment. Interestingly, ALDH1A expression was shown to be inducible by BRD4 and by STAT3 at transcriptional level, however, agents targeting these circuitries may have other non-specific effects and could affect stemness phenotype through non-ALDH related mechanisms.

Here, we tested CM37, a first in class small molecule, discovered as the lead compound from a high-throughput screen of the ChemDiv library. While the compound was found to be potent at nanomolar concentrations in vitro and highly selective for ALDH1A1, its limited bioavailability and short half-life in vivo (~2 minutes) lessened its activity in vivo. Importantly, the inhibitor was tolerated in vivo at concentrations up to 20 mg/kg, without significant systemic toxicity. Efforts to improve its properties are ongoing by using rational chemistry design strategies specifically targeting the “arms” that extend from the central scaffold in order to improve the metabolic stability and modify the lipophilicity of the compound. The observed spectrum of activity of CM37 is in line with reports testing other inhibitors in this class. For example, disulfiram, a broad ALDH inhibitor, was shown to eradicate paclitaxel-resistant triple negative breast cancer cells [24] as well as kinase inhibitor-tolerant lung cancer cells addicted to specific mutant oncogene drivers [30]. However, disulfiram is non-specific and has high systemic toxicity, limiting its potential clinical applicability. Therefore, development of more selective inhibitors has garnered interest. A quinoline-based series of analogues which block ALDH1A1 has been recently described. The lead compound had activity similar to CM37 in OC spheroids and promoted synergy with paclitaxel [31]. Optimization of other novel chemical structures inhibiting ALDH1A1, A2, and A3 has been reported [32]. The lead compound was shown to deplete CSCs, synergize with platinum, and have in vivo activity when injected intra-tumorally [32]. These emerging results point to growing interest exploring this pathway, which is clearly significant to the survival of drug-resistant cancer cells.

The mechanism by which ALDH inhibitors target CSCs remains not fully understood. Here we show that CM37 induces accumulation of ROS, which in turn, causes DNA damage. A disturbance in the intracellular redox balance was also shown to be induced by disulfiram in drug-tolerant lung cancer cells [30] and is consistent with the known functions of the target enzyme. Knockdown of *ALDH1A1* by siRNA was shown to induce DNA damage in another report, consistent with our findings [22]. Thus, we predict that sensitivity to ALDH inhibitors is likely be influenced by the selective expression of ALDH isoforms active in specific contexts and/or by the presence of other mechanisms that regulate oxidative stress in parallel. We cannot exclude that CM37 and other ALDH inhibitors may block stemness through other pathways (e.g., differentiation pathways). Collectively, our results consolidate the concept that ALDH1A1 plays an important role regulating stemness in ovarian cancer, describe the activity of a novel inhibitor targeting this rare and resistant cell population, and support continued efforts to optimize this new class of anti-cancer agents.

## 4. Materials and Methods

### 4.1. Chemicals and Reagents

CM37 was synthesized in the Chemical Genomics Medicinal Chemistry Core at Indiana University School of Medicine, Indianapolis. Trolox was purchased from Sigma-Aldrich (St. Louis, MO, USA). The ALDEFLUOR ALDH activity assay kit was purchased from StemCell Technologies (Vancouver, BC, Canada). Antibodies against phospho-histone H2AX (mAB #9718) and histone H2AX (2595S) were purchased from Cell Signaling Technology (Danvers, MA, USA), and against GAPDH from Biodesign International (Saco, ME, USA). The secondary HRP-conjugated antibodies were purchased from Amersham Biosciences (San Francisco, CA, USA) and Santa Cruz Biotechnology Inc. (Santa Cruz, CA, USA). siRNAs targeting the ALDH1A1 isoform and non-targeting control siRNA were purchased from Dharmacon (Lafayette, CO, USA). Lentiviral particles encoding shALDH1A1 or scrambled, non-silencing control (Sh-control) siRNA were obtained from Sigma-Aldrich.

### 4.2. Cell Lines

The human OC cell lines COV362 and OVCAR5 were provided by Dr. Kenneth Nephew, (Indiana University). SKOV3, OVCAR3, and OV90 OC cell lines were purchased from American Type Culture Collection (Rockville, MD, USA). Cell lines were authenticated using Short Tandem Repeat (STR) DNA profiling analysis and tested mycoplasma negative (IDEXX Bioresearch, Columbia, MO, USA). SKOV3 and primary cells recovered from tumors or ascites were cultured in 1:1 MCDB 105 and M199 (Cellgro, Manassas, VA, USA) supplemented with 10% FBS (Cellgro) and 100 units/mL penicillin and 100 µg/mL streptomycin. OVCAR3 were cultured in DMEM high glucose medium supplemented with 10% FBS and 100 units/mL penicillin and 100 μg/mL streptomycin. OVCAR5 cells were grown in DMEM (high glucose), 10% (FBS), 0.1 mM non-essential amino acids (NEAA), 2 mM L-glutamine, and antibiotics. Spheroid cultures were maintained in Mammocult Complete medium (StemCell Technologies) and ultra-low attachment plates. Cells were cultured at 37 °C in a humidified incubator with 5% CO_2_ supply.

### 4.3. Patient-Derived Primary Human OC Cells

De-identified malignant ascites fluid was obtained from subjects with high-grade serous OC or with primary peritoneal carcinomatosis. The samples were obtained at the Indiana University Simon Cancer Center (IUSCC) and Northwestern University Robert H. Lurie Cancer Center under IRB approved protocols (Indiana University CRO#505; Northwestern University #STU00202468, respectively). For primary cell isolation from human specimens, we used previously described methods [21]. In brief, freshly collected ascites specimens were centrifuged at low speed for 20 minutes. After removal of the fluid, cell pellets were dispersed mechanically and dissociated by using Cellstripper (Corning, Corning, NY, USA; Cat# 25-056-CI). Red blood cell (RBC) (BioLegend, San Diego, CA; Cat#420301) lysis buffer was used to remove RBC and DNase (Qiagen, Valencia, CA, USA; Cat# 79254) was used to digest free DNA in cell suspensions. The single cell suspensions were filtered through a 40 μm cell strainer (ThermoFisher Scientific, Waltham, MA, USA; Cat#NC0147038) to remove other debris before plating in non-adherent plates.

### 4.4. In Vivo Xenograft Studies

*Foxn1nu* nude mice were purchased from Harlan (Indianapolis, IN, USA), and were maintained at the Northwestern University Center for Comparative Medicine in accordance with the National Institutes of Health guidelines and following protocols approved by the Northwestern University Animal Use and Care Committee (Protocol #IS00003060). In brief, 20,000 OVCAR3 OC cells stably transfected with sh-control or sh-ALDH1A1 lentivirus were subcutaneously injected into the flanks of 6–8-week-old female nude mice. Tumors were assessed and measured twice weekly to assess tumor initiation. Time to tumor initiation was determined as the time from inoculation to the time when tumors were detectable by palpation (measuring at least 2 mm in greatest dimension). Tumors were allowed to form and grow until they reached 1500 mm^3^. At that point, mice were euthanized and tumors were harvested, measured, and weighed.

### 4.5. Sphere Formation Assay

The sphere formation assay was performed as previously described, with some modifications [21,25]. Briefly, OC or primary cells were seeded at a density of 10^4^/100 μL in complete MammoCult media supplemented with 1.5% penicillin/streptomycin (Stemcell Technologies, Cambridge, MA, USA) in ultra-low attachment plates (Corning, Corning, NY, USA) and allowed to form spheroids for 7 days. Images were captured, and the numbers of spheres/well were quantified. The data are presented as means ± SEM of triplicate, independent experiments.

### 4.6. ALDEFLUOR Assay and Fluorescence-Activated Cell Sorting

ALDH1 enzymatic activity was measured using the Aldefluor assay kit, per manufacturer recommendations (Stemcell Technologies). Briefly, OC monolayers were dissociated with trypsin and resuspended in Aldefluor assay buffer at a density of 2 × 10^6^ cells/mL. The cell suspension was treated with 5 μL/mL bodipyaminoacetaldehyde (BAAA) and incubated in a 37 °C water bath for 60 minutes. The control sample (ALDH-negative cells), was derived from a 500 μL aliquot of BAAA-treated cell suspension incubated with a 15–30 μM of the ALDH inhibitor, diethylamino benzaldehyde (DEAB), and incubated as mentioned above. As such, the ALDH1A1-positive population was identified and gated using DEAB-treated cells as our control sample. The relative increase in FITC signal of the ALDH-positive cells was determined by a FACS Aria II flow cytometer (BD Biosciences, San Jose, CA, USA) and analyzed in at least two to three independent experiments.

### 4.7. CCK-8 Colorimetric Assay

The numbers of live cells were assessed by using the CCK-8 assay and following the manufacturer’s specifications (Dojindo Molecular Technologies, Rockville, MD, USA). Briefly, OC cells were seeded at a density of 10^4^/100 μL in complete MammoCult media supplemented with penicillin/streptomycin (Stemcell Technologies, Cambridge, MA, USA) in 96-well ultra-low attachment plates (Corning, Corning, NY, USA). Numbers of live cells were assessed after 7 days by adding 10 μL of CCK-8 reagent to each well. Absorbance at 450 nm was quantified using a BioTek plate reader (Winooski, VT, USA). Assays were performed in at least four replicates. Data are presented as means ± SEM.

### 4.8. Cell Titer-Glo Cell Viability Assay

Spheroids were cultured as mentioned above. Numbers of live cells was measured by using Cell Titer-Glo kit (Promega, Madison, WI, USA) per the manufacturer’s recommendations with some modifications. Briefly, an equal volume of CellTiter- Glo reagent was added to each well and the plate was covered with aluminum foil and incubated on an orbital shaker for 30 min at RT. Lysates were transferred to opaque, low-binding polystyrene microplates (Corning, Corning, NY, USA) and allowed to equilibrate for 10 min. ATP abundance was quantified in a SpectraMax GeminiXS luminescence/fluorescence plate reader (Molecular Devices, Palo Alto, CA, USA). Experiments were performed in quadruplicate and repeated at least twice. Data are presented as means ± SEM.

### 4.9. Quantitative Reverse Transcription-Polymerase Chain Reaction (qRT-PCR)

Total RNA was extracted using RNA STAT-60 Reagent (Tel-Test Inc., Friendswood, TX, USA) and reverse-transcribed using an iScript cDNA kit (Bio-Rad, Hercules, CA, USA). Real-time PCR was used to measure *ALDH*, *NANOG*, *OCT4*, *SOX2*, *NIEL3*, *RAD2*, *RAD9A*, *RAD9B*, *RAD51*, and *RAD23A* expression, and human glyceraldehyde-3-phosphate dehydrogenase (*GAPDH*), or beta-actin (*ACTB*/*β-actin*) were used as references. The relative expression of target genes was calculated using the ΔΔCt method. Results are presented as the means ± SD of at least triplicate experiments. Primers are listed below (Table 1).

### 4.10. DNA Damage Pathway PCR-Array

The human DNA Damage Pathway RT^2^ Profiler PCR Array and RT^2^ Real-Timer SyBR Green/ROX PCR Mix were purchased from Qiagen (Valencia, CA, USA). PCR was performed on an ABI Prism 7900 Sequence Detector (Applied Biosystems, Waltham, MA, USA). Data analysis was performed based on the ΔΔCt method with normalization of the raw data to the housekeeping genes, included in the array, using a Microsoft Excel algorithm provided by the manufacturer. For each gene, fold-changes were calculated as the difference in gene expression between control and CM37-treated spheroid cultures. An ontology classification assignment for each gene was performed, and fold-changes were calculated and expressed as percent of composition for each represented pathway in control versus treated spheres.

### 4.11. ALDH Enzymatic Activity

Inhibitory activity of CM37 against ALDH orthologs was tested by using purified recombinant human ALDH1A1, ALDH1A2, ALDH1A3, ALDH2, and ALDH3A1. Dehydrogenase activity of ALDH1A1, ALDH1A2, ALDH1A3, and ALDH2 were measured in a solution containing 100–200 nM enzyme, 200 μM NAD^+^, 1% DMSO, and 100 μM propionaldehyde in 50 mM sodium BES, pH 7.5. ALDH3A1 activity was measured using 25 nM enzyme, 200 μM NAD^+^, 1% DMSO, and 1 mM benzaldehyde in 100 mM sodium phosphate buffer, pH 7.5. All assays were performed at 25 °C and were initiated by the addition of the aldehyde substrate following pre-incubation with compound and NAD^+^. The values represent the average of three independent experiments. Data are presented as percent inhibition.

### 4.12. Immunofluorescence (IF)

OVCAR5 and SKOV3 OC cells were seeded at a density of 5 × 10^4^/mL in complete MammoCult media supplemented with penicillin/streptomycin (Stemcell Technologies, Cambridge, MA, USA) in 6-well ultra-low attachment plates (Corning, Corning, NY, USA) for 5 days and treated with CM37 (1 μM) or DSMO every other day. OC spheroids were fixed, permeabilized, and stained with γH2AX antibody (1:200, Cell Signaling Technology, Danvers, MA, USA). Spheroids were counterstained with Alexa fluor-488 anti-rabbit secondary antibody (1:1000; Molecular Probes, Eugene, OR, USA), and nuclei were visualized by Hoechst staining (Molecular Probes). Images were captured using a Nikon A1 Confocal Laser Microscope at the Northwestern University Center for Advanced Microscopy (Chicago, IL, USA).

### 4.13. Western Blot

Cells were lysed in buffer containing leupeptin (1 μg/mL), aprotinin (1 μg/mL), phenylmethylsulfonyl fluoride (PMSF; 400 μM), and sodium orthovanadate (Na_3_VO_4_; 1 mM). Lysates were briefly sonicated, then cleared by centrifugation (8000× *g*, 10 min, 4 °C), and total protein was measured using the Pierce BCA protein assay kit (ThermoFisher Scientific, Waltham, MA, USA). The lysates were resolved by SDS-PAGE (10% Tris-HCl polyacrylamide gels, Biorad, Hercules, CA, USA) and transferred to PVDF membranes (GE Healthcare Life Sciences, Pittsburg, PA, USA). The membranes were rinsed in Tris-buffered saline with 0.1% Tween 20 (TBS-T) and blocked for 30 min in TBS-T with 5% bovine serum albumin (BSA). Membranes were incubated with primary antibody overnight at 4 °C, and then with secondary antibody for 1 h. Antigen–antibody complexes were visualized using the enhanced chemiluminescence kit (Thermo Scientific, Waltham, MA, USA), and images were obtained using the ImageQuant LAS 4000 mini-imager (GE Healthcare, Pittsburgh, PA, USA). Images were quantified by densitometry using ImageJ32 software (National Institutes of Health, Bethesda, MD, USA). All values were corrected for the integrated optical density (iOD) and normalized to loading controls.

### 4.14. ROS Activity Assay

Mean reactive oxygen species (ROS) accumulation was measured by the DCFDA/H2DCFDA-Cellular Reactive Oxygen Species Detection Assay Kit (Abcam, Cambridge, MA, USA). Briefly, OC cells were resuspended at a concentration of 10^4^ cells/mL in complete MammoCult media supplemented with penicillin/streptomycin (Stemcell Technologies, Cambridge, MA, USA) in 6-well ultra-low attachment plates (Corning, Corning, NY, USA) and allowed to form spheroids for 48 h. Spheroids were dissociated using trypsin, rinsed with complete media (DMEM + 10FBS, + Pen/Strep), then resuspended in complete MammoCult media supplemented with penicillin/streptomycin. Cell suspensions were treated with 20 µM DCFDA and incubated at 37 °C for 30 minutes, then treated with the indicated doses of CM37 or DSMO for 30 min–1 h, depending on the cell line. Positive control samples were treated with 100 µM TBHP. 1 µM propidium iodide was added for cell death exclusion immediately preceding mean ROS measurement by flow cytometry. Data are presented as means ± SEM of independent, triplicate experiments.

## 5. Conclusions

Here we describe the activity of CM37, a new specific and potent small molecule inhibitor for ALDH1A1 in ovarian cancer models enriched in cells with stemness characteristics. Together with siRNA-mediated knockdown of the enzyme, the results obtained using CM37 provide proof of principle supporting the role of ALDH1 in cancer stemness. Our data demonstrate that by fine tuning the levels of intracellular oxidative stress, ALDH1A1, protects cancer cells from DNA damage, enhancing spheroid proliferation and tumorigenicity.

## Figures and Tables

**Table 1:** Real-time PCR Primers.

Target Gene	Forward Primer (5′to 3′)	Reverse Primer (5′to 3′)
*18S*	ACCCGTTGAACCCCATTCGTGA	GCCTCACTAAACCATCCAATCGG
*NANOG*	GATGCCTCACACGGAGACT	TTTGCGACACTCTTCTCTGC
*Oct4*	CTTCGCAAGCCCTCATTTC	GAGAAGGCGAAATCCGAAG
*SOX2*	TGCTGCCTCTTTAAGACTAGGAC	CCTGGGGCTCAAACTTCTCT
*ALDH1A1*	AGGGGCAGCCATTTCTTCTCA	CACGGGCCTCCTCCACATT
*NEIL3*	TCAGAAACTCAATGGAAAGC	CAATACGTTCTGATCCATTAGC
*RAD21*	CAGACTACTGAAGCTCTTTAC	TCCTCCTTTCCTCTTTTTC
*RAD23A*	GGAGAAAGAAGCTATAGAGAGG	CTTTCATGGAATAAGGGTAGG
*GAPDH*	GATTCCACCCATGGCAAATTCC	CACGTTGGCAGTGGGGAC
*RAD9A*	TGTCTTGGCCACACTCTCAG	TGTCTTGGCCACACTCTCAG
*RAD 51*	GAGACCGAGCCCTAAGGAGA	TTAGCTCCTTCTTTGGCGCA
*RAD9B*	GCCTGCTTTTTAGGGCGGAT	ACAATGGCATCAGCAAGCAA

## Supplementary Materials

The following are available online at https://www.mdpi.com/2072-6694/11/4/502/s1, Figure S1: ALDH1A3 expression in cells transfected with sh-RNA targeting ALDH1A1 or scrambled shRNA.
