# Supplementary material for: A Novel ALDH1A1 Inhibitor Targets Cells with Stem Cell Characteristics in Ovarian Cancer
Source: Cancers (Basel). 2019 Apr 8;11(4):502. doi: 10.3390/cancers11040502 (PMC6521036; doi:10.3390/cancers11040502)
Supplement: Supplementary file 1 [file cancers-11-00502-s001.pdf]

cancers

Supplementary Materials

A Novel ALDH1A1 Inhibitor Targets Cells with Stem Cell Characteristics in Ovarian Cancer

Nkechiyere G. Nwani, Salvatore Condello, Yinu Wang, Wendy M. Swetzig, Emma Barber, Thomas Hurley and Daniela Matei

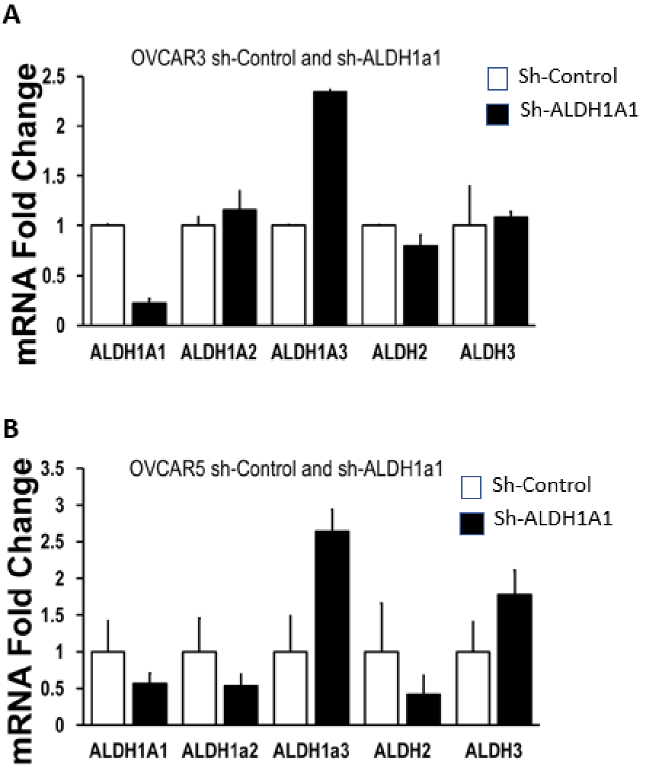

Up/Down Regulation (Compared to Control Group)	
**Symbol**	**1µM CM37**
NEIL3	2.98
RAD21	10.8
RAD23a	8.01

Figure 1. ALDH1A3 expression in cells transfected with sh-RNA targeting ALDH1A1 or scrambled shRNA: (**A**,**B**) qRT-PCR was used to assess ALDH isoform expression levels in OVCAR3 (**A**) and OVCAR5 (**B**) cells stably transfected with sh-ALDH1A1 viral particles.

© 2019 by the authors. Licensee MDPI, Basel, Switzerland. This article is an open access article distributed under the terms and conditions of the Creative Commons Attribution (CC BY) license (http://creativecommons.org/licenses/by/4.0/).
